# The relation between face-emotion recognition and social function in adolescents with autism spectrum disorders: A case control study

**DOI:** 10.1371/journal.pone.0186124

**Published:** 2017-10-11

**Authors:** Anne Lise Høyland, Terje Nærland, Morten Engstrøm, Stian Lydersen, Ole Andreas Andreassen

**Affiliations:** 1 Regional Centre for Child and Youth Mental Health and Child Welfare, Norwegian University of Science and Technology, Trondheim, Norway; 2 Department of Pediatrics, St. Olavs Hospital, Trondheim University Hospital, Norway; 3 NevSom, Department of Rare Disorders and Disabilities, Oslo University Hospital, Norway; 4 NORMENT, KG Jebsen Centre for Psychosis Research, University of Oslo, Oslo, Norway; 5 Department of Neurology and Clinical Neurophysiology, St. Olavs Hospital, Trondheim University Hospital, Norway; 6 Department of Neuroscience, Norwegian University of Science and Technology, Trondheim, Norway; 7 Division of Mental Health and Addiction, Oslo University Hospital, Oslo, Norway; McGill University, CANADA

## Abstract

An altered processing of emotions may contribute to a reduced ability for social interaction and communication in autism spectrum disorder, ASD. We investigated how face-emotion recognition in ASD is different from typically developing across adolescent age groups. Fifty adolescents diagnosed with ASD and 49 typically developing (age 12–21 years) were included. The ASD diagnosis was underpinned by parent-rated Social Communication Questionnaire. We used a cued GO/ NOGO task with pictures of facial expressions and recorded reaction time, intra-individual variability of reaction time and omissions/commissions. The Social Responsiveness Scale was used as a measure of social function. Analyses were conducted for the whole group and for young (< 16 years) and old (≥ 16 years) age groups. We found no significant differences in any task measures between the whole group of typically developing and ASD and no significant correlations with the Social Responsiveness Scale. However, there was a non-significant tendency for longer reaction time in the young group with ASD (*p* = 0.099). The Social Responsiveness Scale correlated positively with reaction time (*r* = 0.30, *p* = 0.032) and intra-individual variability in reaction time (*r* = 0.29, *p* = 0.037) in the young group and in contrast, negatively in the old group (*r* = -0.23, *p* = 0.13; *r* = -0.38, *p* = 0.011, respectively) giving significant age group interactions for both reaction time (*p* = 0.008) and intra-individual variability in reaction time (*p* = 0.001). Our findings suggest an age-dependent association between emotion recognition and severity of social problems indicating a delayed development of emotional understanding in ASD. It also points towards alterations in top-down attention control in the ASD group. This suggests novel disease-related features that should be investigated in more details in experimental settings.

## Introduction

Autism spectrum disorder (ASD) is a neurodevelopmental disorder characterized by a reduced ability to participate in social interactions and a tendency to engage in repetitive and stereotypic behaviors [1]. Recent research on the neurobiology of ASD has provided insight into the genetic basis [2], the brain abnormalities [3, 4] and the cognitive aspects of the impairments [5]. Deficits in emotional understanding are identified as one of the diagnostic criteria for ASD in both the Diagnostic and Statistical Manual of Mental Disorders (DSM-5, American Psychiatric Association 2013) and the International Statistical Classification of Diseases and Related Health Problems, (ICD-10, World Health Organization 2004). Face-emotion recognition precedes emotional understanding and studies applying pictures of facial emotional expressions suggest that abnormalities in emotion recognition may underlie some of the social difficulties associated with ASD [6]. Furthermore, it seems like age is of importance in emotional understanding [7]. An interesting hypothesis is that deviant development trajectories underlie the face processing impairments in individuals with ASD [8].

The development of emotional understanding has been extensively studied. The ability to recognize faces is present in infants [9] and seems to be further developed through childhood and adolescence due to interactions between social stimuli and the neurobiology of “social brain circuits” [10]. Lawrence et al. [7] explored the developmental trajectory of emotion recognition in typically developing (TD) children between the ages of 6 and 16 years. They found a significant age effect in the ability to recognize happiness, surprise, fear and disgust. With respect to sad and angry faces, six-year-old children demonstrated near-adult levels of accuracy. Tonks et al. [11] assessed emotion recognition in TD children between the ages of 9 and 15 years and found that they hardly improved emotion recognition after the age of 11 (ceiling effect). Behaviors related to social cognition dramatically change during adolescence, and this is paralleled by functional changes in the social areas of the brain [12]. Deviant emotional processing seems also to play a role in ASD development, but here the evidence is less clear. Peterson et al. [13] found that children with ASD up to 12 years of age experienced greater difficulty reading emotions from the eyes than TD. Kuusikko et al. [14] described reduced capabilities to recognize emotions, especially anger. Their findings support the notion that both children and adolescents with ASD have difficulties recognizing emotions and that this ability improves with age. Other findings also suggest delayed ability of face recognition in ASD [8]. There was no time pressure in any of these studies and we have limited knowledge about how different age groups with ASD understand rapidly changing, steady upcoming facial expressions.

Understanding of emotions requires interpretation of facial expressions [15]. Results of studies of face-emotion recognition are inconsistent in ASD and the diverging results may be due to the type of paradigm used [16]. A key challenge is related to the method for presenting the facial expression. Clark et al. (2008) argues that the duration of exposure of the pictures in the studies affects the results. Short presentation times demand a holistic strategy of facial recognition rather than focus on details [17], whereas an altered mechanism for emotion recognition in ASD may contribute to the social difficulties [18]. When pictures of faces were presented for a brief period of time, adults with ASD (mean age 26 years) were found to be significantly less accurate than TD (mean age 19,6 years) [6]. Pictures of emotional faces presented for 80 milliseconds (ms) with inter-stimulus time of 1300–1500 ms also revealed significant differences between ASD and TD adolescents (mean age 14 years) in magnetoencephalography recordings indicating atypical neuronal activity in ASD [19].

Emotional processing is closely linked to social interaction [20]. Social orienting is a prerequisite for social development, and the social motivation theory of ASD has recently gained new interest [21]. It seems likely that the ability to rapidly extract and interpret emotions (emotional processing) impacts social-emotional function and interpersonal reciprocity and thereby social motivation [22]. Some studies show that persons with high functioning ASD have normal ability to categorize basic facial emotions [23]. ASD show difficulties in recognition of complex emotions [24]. Longer reaction times were shown for individuals with ASD when identifying facial expressions presented in a continuum of changing emotions [25]. However, the relationship is still not well understood. There may be other factors explaining the differences in the literature. Variation in disease severity and IQ may contribute to the different findings [16].

In addition to reaction time on a face-emotion recognition task, recent evidence suggests that intra-individual variability (IIV) may distinguish ASD from other developmental disorders and TD [26]. Lundervold et al. [26] found less variability in the ASD group compared to ADHD, combined ASD and ADHD and TD using Conners’ Continuous Performance Test (CPT-II) [27]. Vaurio et al.[28] reported increased IIV with increased cognitive load. However, no study has investigated how variability changes with age in ASD. The visual CPT (VCPT) has been used in studies with neuropsychiatric disorders such as ADHD [29], but not yet in ASD and there have been few attempts to compare the differences when using neutral or emotional stimuli in a cued GO/ NOGO paradigm. Since emotional processing seems affected in ASD, it would be of interest to investigate differences between a standard VCPT paradigm and VCPT with emotional pictures, ECPT [30].

The aim of the current study was to investigate aspects of emotion processing in ASD focusing on rapid and fluent recognition of facial emotions in different age groups of adolescents with ASD compared to TD. We also aimed to determine the relation between ECPT performance and social functioning measured by the SRS. We applied a novel paradigm presenting the stimuli as a continuous load of brief pictures in a cued GO/ NOGO paradigm using pictures of emotional faces, ECPT. This design requires both emotion recognition, attention orienting and inhibition control. We hypothesized that the time to extract emotions, reaction time (RT ECPT), is increased in the adolescents with ASD compared to the TD group. We expected these abnormalities to be associated with core ASD symptoms in social functioning and consistent through our age span. Because the evaluation time for each stimulus was limited, we expected the ASD group to fail more often in the recognition of emotions. As understanding of emotions and motivation for social interaction are both crucial for social function, we also investigated the relations between RT ECPT and the subscales Social Cognition and Social Motivation in the SRS. Reduced IIV is reported in ASD [26], and we also investigated the IIV in relation to diagnosis, severity of social symptoms and age group.

## Methods

### Participants

Fifty adolescents with a prior diagnosis of ASD without intellectual disability from outpatients attending St. Olavs Hospital, Trondheim, Norway, were included in the study (Table 1).

**Table 1:** Demographics.

	ASD		TD	
	*n*	%	*n*	%
	49	100	49	100
**Gender**				
Male	36	73.5	31	63.3
Female	13	26.5	18	36.7
**ASD subgroup**				
Infantile autism	13	26.5		
Asperger disorder	18	36.7		
PDD NOS	18	36.7		
**Age–years**				
Mean (*SD*); range	15.6 (±2.4); 11.9–20.9		15.6 (±1.8); 12.3–19.4	
< 16 years	26	53.1	27	55.1
Mean (*SD*); range	13.7 (±1.3); 11.9–15.7		14.2 (±1.0); 12.3–15.7	
≥ 16 years	23	46.9	22	44.9
Mean (*SD*); range	17.8 (±1.3); 16.1–21.0		17.3 (±1.1); 16.1–19.4	
**IQ** Mean (*SD*); range				
Full scale IQ (*n* = 36)	91.9 (±17.7); 67–133			
Verbal IQ (*n* = 47)	87.6 (±19.0); 52–130			
Nonverbal IQ (*n* = 48)	98.1 (±19.3); 58–139			
**Comorbidity**				
No comorbidity	31	63.3		
More than one comorbidity	7^1^	14.3		
Comorbid AD/HD	17^1^	34.7		
**SCQ** Mean (*SD*); range	18.7 (±6.7); 5–34		1.9 (±2.3); 0–8	
**SRS** Mean (*SD*); range	80.1 (±14.4); 47–109		40.6 (±4.2); 34–51	

^1^ All but one participant with comorbidity had comorbid AD/HD. These are hence reported twice in the table, both in “More than one comorbidity” and “Comorbid AD/HD”.

Separate information for each diagnostic group (S1 Table) and for each age group (S2 Table) is available in Supporting Information.

The participants were between 12 and 21 years with 13 girls and 37 boys. The ASD individuals were diagnosed according to the ICD-10 F.84 criteria for pervasive developmental disorder based on developmental information and clinical assessments. They were also sub-grouped into infantile autism, Asperger’s syndrome and pervasive developmental disorder, unspecified, PPD-NOS. The ADOS (Autism Diagnostic Observation Schedule [31]) was used in 43 of 50 cases. IQs were obtained in the ASD group. Full-scale IQs (FIQs) ranged between 67 and 133. When the difference between verbal and performance IQs was ≥ 30, we did not calculate FIQs. To be included in the study, verbal or performance IQ had to be within the normal variation (≥ 70). Eighteen (37%) individuals in the ASD group had neuropsychiatric comorbidity, all but one with attention problems (Attention Deficit Disorder with or without hyperactivity (AD/HD)). The differences in comorbidity with AD/HD in the younger versus older is not significant (Pearson’s chi squared, *p* = 0.234). Eight (16%) had more than one comorbid diagnosis. Six (12%) had an epilepsy diagnosis, all but one with co-occurring AD/HD. All participants had a six-minute resting EEG registration. A specialist in clinical neurophysiology examined the registrations and found no epileptic activity. Twelve (25%) of the ASD individuals received medication. Four were on stimulants, two used atomoxetine and the six with epilepsy were on antiepileptic medication.

We recruited 49 TD adolescents, matched for age and gender as a control group. These individuals were recruited from adjacent schools through invitations/bulletins to all students/parents. Also, the parents were involved, and in writing confirmed that their child did not suffer from any chronic disease or psychiatric problems now or previously.

To investigate developmental differences, we divided the participants into two age groups. We split at the age of 16 years to obtain equal group sizes. The < 16-years group (young group) included 27 ASD individuals and 27 TD, and the ≥ 16-years group (old group) included 23 ASD individuals and 22 TD.

One of the participants in the ASD young group scored > 70% inattention on the continuous performance test and was excluded. The others, 49 ASD individuals and 49 TD, were included in the study.

### Measures

#### Cued GO/NOGO task

**Fig 1:**
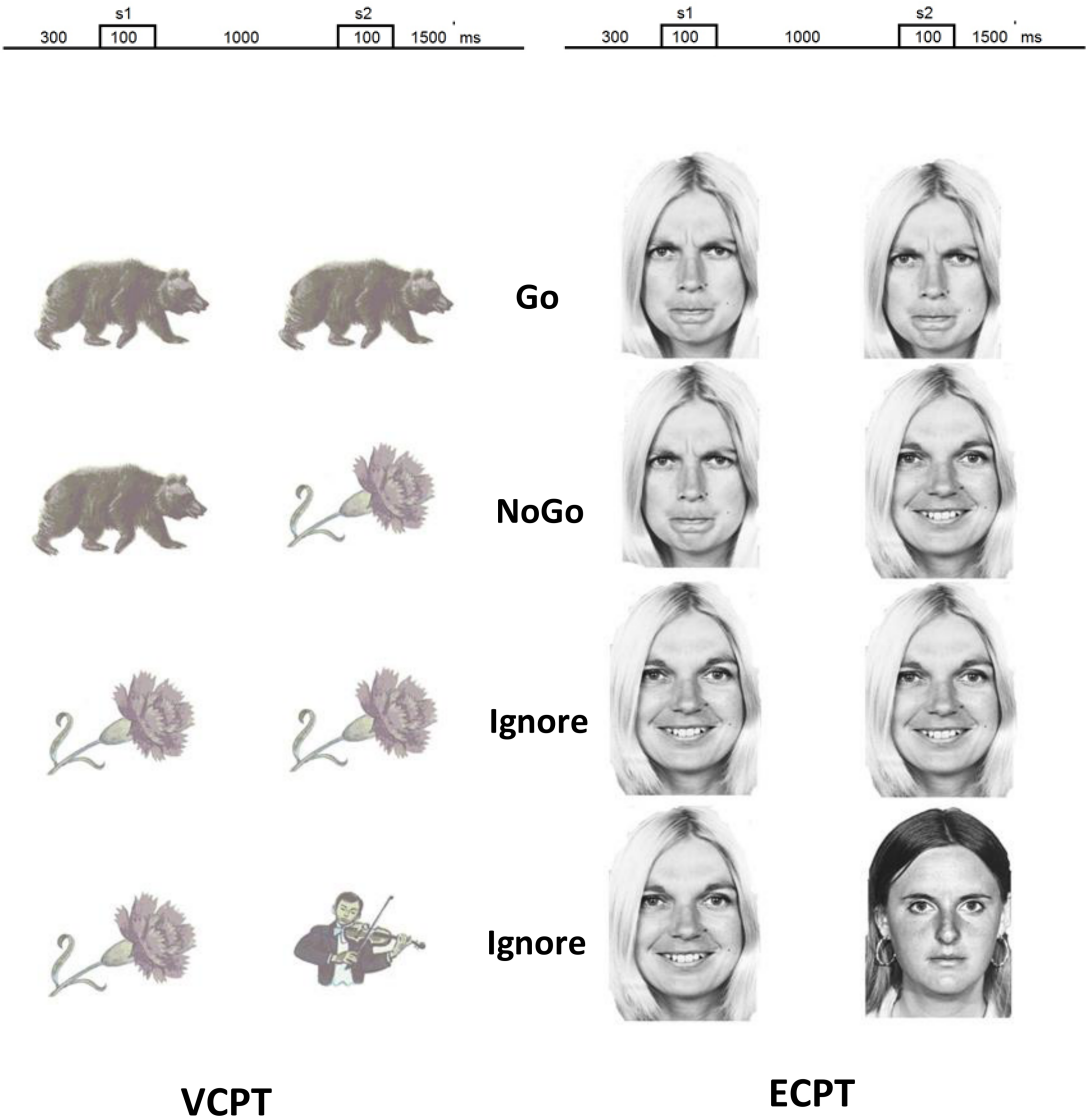
Task stimuli, VCPT and ECPT.

The Visual Continuous Performance Test (VCPT) measures variables of attention and reaction time using a cued GO/ NOGO task [29]. The three categories of visual stimuli include 15 pictures of animals, 15 pictures of plants and 15 pictures of humans (Fig 1). The Emotional Continuous Performance Test (ECPT) is a similar test as the VCPT but uses pictures of faces with emotional affect [32] from Ekman and Friesen [33]. The categories of the pictures on the ECPT include 15 pictures of angry faces, 15 pictures of happy faces and 15 pictures of neutral faces (Fig 1). The trials present pairs of pictures: animal–animal on the VCPT or angry–angry on the ECPT (GO trials), animal–plant/ angry–happy (NOGO trials), plant–plant/ happy–happy and plant–human/ happy–neutral (IGNORE trials). The participants were asked to respond by pressing a button with right index finger as quickly as possible without making mistakes in all GO trials and otherwise refrain from responding. Each trial consists of two pictures presented for 100 ms with an 1100 ms inter-stimulus interval and an inter-trial interval of 3000 ms. The trials for each task (VCPT and ECPT) are grouped into three blocks separated by a short break. In each block, a unique set of five pictures from each picture category is selected. Each block consists of a pseudo-random presentation of 100 stimuli pairs with equal probability for each trial category.

All participants were first presented the VCPT, which was immediately followed by the ECPT. The participants sat in a comfortable chair 1.2 m from the computer screen during the task. The pictures were presented on an 18-inch monitor using the Psytask (http://bio-medical.com/products/psytask.html) software (from Bio-medical, Clinton Township, Michigan USA). The time interval from the presentation of the second stimulus to response was registered by VCPT/ECPT software as the reaction time (RT VCPT/ RT ECPT). The reported reaction time is the average time for correct responses. The intra-individual variability, IIV, measured as Standard Error, $\frac{SD}{\surd n}$, and the number of omissions and commissions (response in NOGO-trials) were also registered.

All participants were tested by the same technician in the same lab to reduce variations caused by testing conditions.

#### Social Communication Questionnaire (SCQ) and Social Responsiveness Scale (SRS)

The ASD diagnosis was supported by the Social Communication Questionnaire (SCQ) [34]. The SCQ is a 40-item parent report questionnaire based on the Autism Diagnostic Interview–Revised (ADI–R; Lord et al. 1994) and is validated for the diagnosis of autism (Berument et al. 1999). The autistic symptom severity was measured by the Social Responsiveness Scale (SRS) [35]. SRS is a 65-item questionnaire for caregivers where they quantify the level of autistic traits or autistic severity [36]. The reliability and validity of SRS seems satisfactory [37, 38], and SRS scores are associated with Autism Diagnostic Interview–Revised (ADI–R) scores [39]. It generates scale scores for specific symptom domains as well as a singular total score, which indicates the severity of social impairment [40]. We registered the total score and two subscale scores, Social Cognition and Social Motivation, in both the ASD and the TD.

The ASD group had significantly higher mean symptom scores on both instruments compared to the TD group. (SCQ *p*<0.001 and SRS *p*<0.001). There were no differences in these scores between the different age groups.

### Study design and outcomes

The primary outcome for this study was facial emotion recognition time, i.e. RT ECPT, related to diagnosis and social function measured by SRS total score. This reaction time is influenced by the participants' ability to rapidly and correctly recognize the emotions, but also the individual’s general reaction time. We therefore adjusted our analyses for RT VCPT as an estimate of general reaction time. Subsequently we investigated the relation between RT ECPT and the sub-scales Social Cognition and Social Motivation of the SRS, and the results from the subscales are reported as sub-analyses.

Secondary outcomes were failures of omissions, failures of commissions and intra-individual variability, IIV, measured as the standard error.

Measure scores were analyzed for the whole group of participants and separately within each of the two age groups.

### Statistical analysis

Groups were compared using the Pearson chi squared test for categorical variables and the Student’s *t*-test for continuous variables.

We compared the difference between RT ECPT and RT VCPT using independent *t-*tests and we computed the association between RT ECPT and RT VCPT for all participants using Pearson’s correlation coefficient. We carried out regression analyses with Reaction Time, RT ECPT, as dependent variable, and diagnosis (ASD versus TD), SRS total scale or subscales one at a time as independent variables. These analyses were done for the complete sample adjusted for age group, separately for each age group, and for the complete sample including age group and its interaction with diagnosis, SRS total scale or subscales. All these analyses were adjusted for RT VCPT. Where relevant, we also calculated partial correlations between RT ECPT and diagnosis/ SRS scores adjusting for the same variables. We compared RT ECPT separately for ASD and for TD between the young and old age groups using Student’s *t*-test. We also computed correlations between SRS and subscales and IQ.

The number of failures in ECPT (omissions and commissions) plus one were log transformed to obtain approximate normality. Number of failures and differences in omissions between VCPT and ECPT were compared using Student’s *t*-test.

We then used IIV ECPT and IIV VCPT as dependent variables in linear regression analyses with diagnosis and SRS with subscales, respectively, as independent variables. These analyses were done for the complete sample adjusted for age group, separately for each age group, and for the complete sample including age group and its interaction with diagnosis and SRS. We also computed the partial correlations between these variables adjusting for age group.

We analyzed the correlations between RT ECPT/ VCPT and IIV ECPT/ VCPT and IQ in the ASD group.

Normality of residuals was checked using visual inspection of Q-Q plots. Two-sided *p*-values < 0.05 were considered statistically significant, and 95% confidence intervals were reported where relevant. Due to multiple comparisons, *p*-values between 0.01 and 0.05 should be interpreted with caution. Statistical analyses were carried out in SPSS 24.

## Results

### Reaction time (RT)

#### TD and ASD group comparisons

RT ECPT and RT VCPT, IIV ECPT and IIV VCPT are presented in Table 2.

**Table 2:** Reaction time, RT, for ECPT and VCPT and intra-individual variability, IIV, for ECPT and VCPT; mean ± SD; range. All in milliseconds.

		ASD, *n* = 49	TD, *n* = 49
**RT ECPT**	All	393.9 ± 70.2 (268 to 583)	380.4 ± 54.8 (291 to 532)
	< 16 years	418.4 ±74.2 (301 to 583)	388.4 ±45.1 (316 to 532)
	≥ 16 years	366.3 ± 54.7 (268 to 447)	370.4 ±64.6 (291 to 511)
	All	338.3 ± 65.0 (251 to 542)	330.5 ±62.0 (254 to 559)
**RT VCPT**	< 16 years	346.2 ± 71.5 (251 to 542)	328.9 ±46.4 (271 to 480)
	≥ 16 years	329.4 ± 57.1 (260 to 490)	332.6 ±78.2 (254 to 559)
	All	14.8 ± 5.9 (5.4 to 33.8)	14.5 ± 4.7 (5.4 to 29.8)
**IIV ECPT**	< 16 years	17.4 ± 5.7 (8.9 to 33.8)	15.2 ± 4.4 (8.8 to 29.8)
	≥ 16 years	11.8 ± 4.5 (5.4 to 21.4)	13.6 ± 4.9 (5.4 to 22.8)
**IIV VCPT**	All	9.9 ± 3.6 (3.8 to 20.3)	10.0 ± 3.7 (3.8 to 21.6)
	< 16 years	11.4 ± 3.7 (4.9 to 20.3)	10.7 ± 3.9 (4.1 to 21.6)
	≥ 16 years	8.2 ± 3.3 (3.8 to 17.9)	9.2 ± 3.4 (3.8 to 17.9)

The RT ECTP was significantly longer than RT VCPT both in the TD (*p*<0.001) and in participants with ASD (*p*<0.001). RT VCPT correlated significantly with RT ECPT in TD (*r* = 0.79, *p*<0.001) and ASD (*r* = 0.79, *p*<0.001). There was no significant difference between ASD and TD in RT ECPT adjusted for RT VCPT, see Table 3. We repeated these analyses separately for the two age groups. There were no significant differences in RT ECPT in the two age groups.

**Table 3:** Linear regression with Reaction Time ECPT (RT ECPT) as dependent variable, and diagnosis, SRS total score (primary outcome) and the subscales (sub-analyses) one at a time as independent variables. Complete sample (a), Separate analyses for each age group (b and c), and complete sample including age group and its interaction with diagnosis, SRS total score or subscales (d). All analyses are adjusted for RT VCPT.

Independent variables	Regression coefficient β, (*confidence interval*), *p*
**(a)** ASD vs. TD	7.98(-6.53 to 22.48), *p =* 0.28
SRS total score	0.08(-0.25 to 0.41), *p =* 0.65
SRS–Social cognition	0.03(-0.30 to 0.36), *p =* 0.87
SRS–Social motivation	0.14(-0.25 to 0.53), *p =* 0.48
**(b) < 16 years**	
ASD vs. TD	14.91(-2.91 to 32.73), *p =* 0.099
SRS total score	0.43(0.04 to 0.82), *p =* 0.032*
SRS–Social cognition	0.39(-0.03 to 0.80), *p =* 0.065
SRS–Social motivation	0.66(0.20 to 1.12), *p =* 0.006**
**(c) ≥ 16 years**	
ASD vs. TD	-2.53(-26.20 to 21.13), *p =* 0.83
SRS total score	-0.41(-0.94 to 0.13), *p =* 0.13
SRS–Social cognition	-0.40(-0.92 to 0.11), *p =* 0.12
SRS–Social motivation	-0.52(-1.13 to 0.09), *p =* 0.091
**(d) Interaction with age group**	
ASD vs. TD * age group	*p =* 0.20
SRS total score * age group	*p =* 0.008**
SRS–Social cognition * age group	*p =* 0.014*
SRS–Social motivation * age group	*p =* 0.002**

* Significant at 0.05-level

** Significant at 0.01-level

We computed the age-related differences in RT ECPT separately within the ASD and the TD and found a significant reduction of RT ECPT only in ASD (*p* = 0.008, TD; *p* = 0.26).

#### Relation to degree of social problems

**Fig 2:**
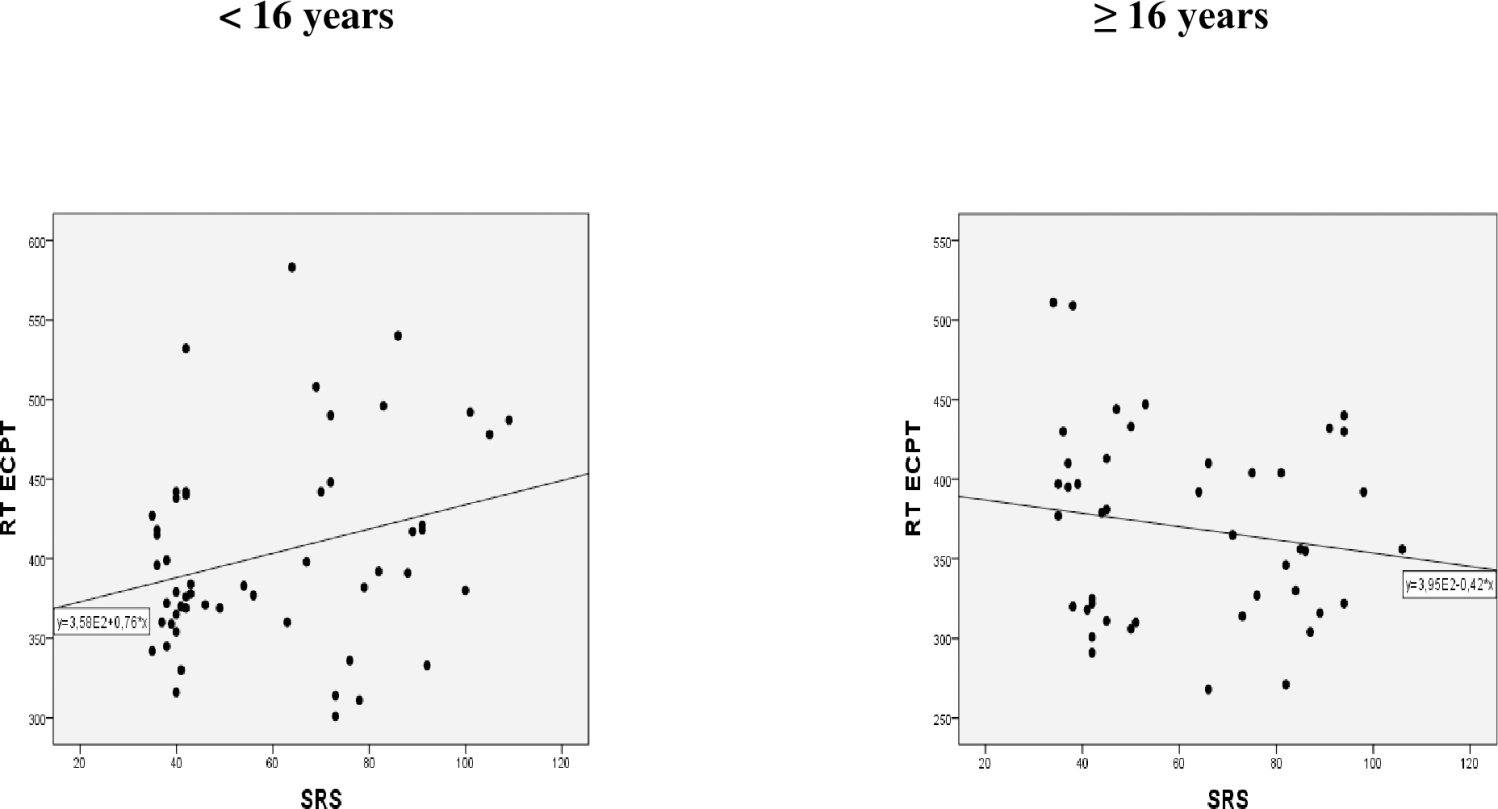
Scatter plots of RT ECPT related to SRS in the two age groups.

No significant association was found between the SRS total score and RT ECPT for the whole group of participants (Table 3, part a). For the young group, the RT ECPT correlated significantly with the SRS total score (*r* = 0.30, *p =* 0.032), see Fig 2. The RT ECPT correlated also significantly with the social motivation subscale (*r* = 0.38, *p =* 0.006) and was borderline significant with SRS social cognition (*r* = 0.26, *p =* 0.065). We did not find significant correlations with SRS total scale or subscales in the old group (SRS total score: *r* = -0.23, see Fig 2, *p =* 0.13; social cognition: *r* = -0.24, *p =* 0.12; social motivation: *r* = -0.26, *p =* 0.091), but note that all correlations in this age group were negative; i.e. in the opposite direction of the young.

When including the interaction between SRS scores and age group in the linear regression, we found significant interactions with SRS total score (*p* = 0.008), social cognition (*p* = 0.014) and social motivation (*p* = 0.002), see Table 3 part (d). We computed correlations between SRS with subscales and IQ without finding significant relations.

### Omissions / Commissions

Number of omissions (mean ± SD) was not significantly different (*t* (96) = -0.4, *p =* 0.73) between the the ASD (7.8 ± 9.7)and the TD groups (5.9 ± 4.9), and errors of commissions were also not different (*t* (96) = 0.8, *p =* 0.42) between ASD (2.5 ± 2.7), and TD (3.2 ± 3.7). The difference in omissions between VCPT and ECPT in the two groups was also non-significant (*t*(96) = 1.5, *p* = 0.15). Separate analysis for each of the two age groups yielded similar results. There were no significant correlations between omissions / commissions and the SRS with subscales.

### Intra-individual variability (IIV)

No significant association was found between IIV ECPT and diagnosis or SRS total score for the whole group of participants (Table 4). However, IIV ECPT and diagnosis correlated in different directions in the age groups (young group *r =* 0.21, *p* = 0.12 and old group *r* = -0.19, *p* = 0.21) giving a significant age group * IIV ECPT interaction (*p* = 0.049). IIV ECPT also correlated significantly with SRS in opposite directions in the two age groups (young group *r =* 0.29, *p* = 0.037 and old group *r* = -0.38, *p* = 0.011)), with a significant interaction between SRS scores and age group (*p* = 0.001). The IIV VCPT correlated non-significantly, but in the same directions as IIV ECPT for the two age groups (young group *r =* 0.11, *p* = 0.43 and old group *r* = -0.21, *p* = 0.18), giving a non-significant interaction, *p* = 0.14. Secondary analyses for adolescents within the different subgroups and with and without comorbidity gave substantially the same main results. Further, for the ASD, we computed correlation analyses for IQ and RT VCPT, RT ECPT, IIV VCPT and IIV ECPT without significant relations.

**Table 4:** Linear regression with intra-individual variability in reaction time, IIV, as dependent variable, and diagnosis and SRS total score (primary outcome) and the subscales (sub-analyses) one at a time as independent variables. Complete sample (a), Separate analyses for each age group (b and c), and complete sample including age group and its interaction with diagnosis or SRS total scale (d).

Independent variables	IIV ECPT Regression coefficient β, (*confidence interval*), *p*	IIV VCPT Regression coefficient β, (*confidence interval*), *p*
**(a)** ASD vs. TD	0.30(-1.83 to 2.43), *p =* 0.78	-0.13(-1.61 to 1.35), *p* = 0.86
SRS total score	0.00(-0.05 to 0.05), *p =* 0.92	0.00(-0.04 to 0.03), *p* = 0.79
SRS–Social cognition	-0.01(-0.06 to 0.04), *p* = 0.72	-0.01(-0.04 to 0.03), *p* = 0.61
SRS–Social motivation	0.00(-0.06 to 0.06), *p* = 0.94	0.00(-0.04 to 0.04), *p* = 0.95
**(b) < 16 years**		
ASD vs. TD	2.20(-0.62 to 5.02), *p =* 0.12	0.74(-1.36 to 2.84), *p* = 0.48
SRS total score	0.07(0.00 to 0.13), *p =* 0.037*	0.02(-0.03 to 0.07), *p* = 0.43
SRS–Social cognition	0.01(-0.06 to 0.04), *p* = 0.06	0.02(-0.03 to 0.07), *p* = 0.42
SRS–Social motivation	0.09(0.01 to 0.16), *p* = 0.021*	0.04(-0.02 to 0.09), *p* = 0.17
**(c) ≥ 16 years**		
ASD vs. TD	-1.78(-4.60 to 1.04), *p =* 0.21	-1.05(-2.91 to 0.81), *p* = 0.26
SRS total score	-0.08(-0.14 to -0.02), *p =* 0.011*	-0.03(-0.07 to 0.01), *p* = 0.18
SRS–Social cognition	-0.07(-0.13 to -0.01), *p* = 0.018*	-0.03(-0.07 to 0.01), *p* = 0.12
SRS–Social motivation	-0.09(-0.16 to -0.016), *p* = 0.017*	-0.04(-0.09 to 0.01), *p* = 0.088
**(d) Interaction with age group**		
ASD vs. TD * age group	*p =* 0.049*	*p =* 0.21
SRS total score * age group	*p =* 0.001**	*p =* 0.14
SRS–Social cognition	*p =* 0.003**	*p =* 0.12
SRS–Social motivation	*p =* 0.001**	*p =* 0.034*

* Significant at 0.05-level

** Significant at 0.01-level

## Discussion

The main findings of the current study were that the younger ASD group (12–16 years) demonstrated a tendency to require more time recognizing facial emotions than the TD. Furthermore, in this age group, enhanced IIV ECPT correlated positively with social problems measured by SRS. In older adolescents (**≥**16 years), there was no difference between the ASD group and the TD, while the reaction time and IIV correlated negatively with social problems. This resulted in a significant age-dependent interaction between RT and IIV ECPT with social problems. Given the high heterogeneity within ASD generally as also reflected in the participants of this study, the findings should be replicated in an independent sample to be sure it is generalizable to ASD in general.

Others have also studied reaction times in recognition of emotion paradigms. The present mean RT ECTP was significantly longer than RT VCPT which is consistent with the findings of Markovska-Simoska and Pop-Jordanova[30]. They suggested that increased RT in ECTP could be due to the influence of emotional stimuli on attention and information-processing. Akechi et al. [41] recorded reaction times after presenting from the same stimuli set as used in our study to children with ASD and TD aged 9 to 14 years. They found no differences in accuracy or reaction time regarding the recognition of emotions. However, their inter-stimulus interval was adjusted to the time the individual needed to respond or at maximum five seconds. This differs significantly from the 1800 ms used in our study, which may explain the divergent results.

The SRS subscale for social motivation correlated positively with RT ECTP among the younger (12–16 years) adolescents in the present study. This finding is consistent with the social motivation theory of autism, which proposes that ASD is an extreme case of diminished social motivation [21]. Social motivation may drive the development of basic skills necessary for appropriate social interaction as interpretation of faces [21]. Lozier et al. [16] found a large and generalized defect in facial emotion recognition in individuals with ASD and that the magnitude of this defect increased with age. In our study, we found the largest difference from TD in the younger group (12–16 years).

After the age of 16, we found no significant differences between TD and ASD in RT ECPT. This suggests that the ASD group was able to obtain normal emotion recognition, but at a later age than the TD. The delayed emotion recognition development in ASD may be due to both biological maturation and cognitive training. Further, TD showed equal RT ECPT in our age groups, supporting that the ability to recognize emotions is established at age 12 years. McGovern and Sigman [42] reported a significant improvement in social function in ASD between mid-school and adolescence measured by the ADI-R. Their different results for the younger and older age groups are consistent with the findings of the current study.

We found differences in the intra-individual reaction time variability (IIV) in the present study. This measure is reported to be a marker for the efficiency of top-down attentional control [43–45]. Vaurio et al. [28] found increased variability with increased cognitive demands. The increased IIV ECPT in young ASD may reflect the difficulty of the task. Karalunas et al. [46] reported conflicting findings in IIV in ASD. During our test procedure, it was observed that participants motivated to complete the test properly had better endurance. It was the impression of the test administrator that the motivation of the TD group decreased faster than in ASD, especially in the old group. Decreased IIV with age in TD is consistent with more stable attention and is in line with earlier findings [47].

In the present study, IIV ECPT was positively correlated with social problems for younger ASD participants (12–16 years), and negatively correlated for the older group (≥ 16 years) resulting in a significant interaction between SRS and age group. The specific underlying mechanisms contributing to these age-dependent results are not known and the current findings should be followed up with more investigations. However, it is possible that an increase in cognitive demands due to difficulties in emotion recognition may underlie the increased IIV ECPT in the younger ASD group. Reduced IIV associated with higher SRS scores in the older ASD group (≥ 16 years) may reflect the observation that TD group seemed less engaged in the task than the ASD individuals.

The emotion recognition task applied in the current study included micro expressions with presentation times below 200 ms. Shen et al. [48] found that micro expressions challenge the ability of emotion recognition in TD individuals. In our study, the presentation time was 100 ms, which we expected would be a challenge for the ASD group. However, the rate of omissions/commissions was not different in the TD and ASD groups. This lack of significant differences may be attributable to low power, as there were trend level differences. Another aspect of the paradigm is related to the use of basic emotions. The participants were only asked to recognize a single basic emotion, anger. They implicitly had to exclude happy to define the “GO-condition”. Previous studies have shown that more complicated and subtle emotional expressions are more difficult for individuals with ASD to recognize than the basic emotions [49]. Thus, this may have reduced the opportunity to find differences in our study.

Social-emotional functions are one of the hallmarks of ASD. Biological abnormalities in the social motivation network may influence the basic premise for social interactions, given that social orienting and attachment are necessary for the continued development of social functioning through childhood and adolescence [21]. Moreover, social interaction requires the rapid processing of emotions to ascertain the intentions, motivations and emotional reactions of other people. Thus, a less immediate understanding of emotions in children with ASD may impact social training [10]. The significant correlation between the SRS subscale social motivation and RT ECPT for the young groups (< 16 years) in the present study supports a relationship between coping and motivation. Targeted training in understanding emotions could improve ASD symptoms. and should be included in treatment programs. Furthermore, interacting socially with peers also provide useful experience. Enhanced emotional understanding in early childhood may increase social interaction and influence the development of abnormal social reciprocity in individuals with ASD.

### Strengths and limitations of the study

Though we included patients previously diagnosed with ASD, we did not repeat the diagnostic assessment. The frequencies of diagnostic subcategories are not balanced between the two age groups. As autistic symptoms also occur to some extent in the population of TD, we addressed this by completing SCQ and SRS in both ASD individuals and TD without revealing significant differences between the diagnostic groups. Therefore, we carried out analyses based on the dichotomous diagnostic groups, as well as based on the autistic symptoms measured through SRS. This enabled the inclusion of all participants in our analyses.

We did not obtain the IQs for the TD, and were thus unable to match the IQs in the healthy control group and individuals with ASD. Cognitive level would be expected to influence both RT and IIV and could bias the results. However, individuals with autism typically have divergent verbal IQs compared to performance IQs which makes it challenging to match a control group. In the invitation letter and recruitment posts we specifically invited healthy adolescents. Thus, adolescents with learning difficulties such as dyslexia were not motivated to participate. Also, the parents were involved, and in writing confirmed that their child did not suffer from any chronic disease or psychiatric problems presently or previously. Due to the recruitment procedure, we expected most of the participants to have IQ in the normal range.

Another limitation may be the lack of randomization of VCPT vs ECPT order. The VCPT and ECPT are reported to have different performance measures, with RT and IIV increased in ECPT compared to VCPT. This is attributed to the emotional content of the ECPT-pictures. Our main objective was to compare the differences in performance between ASD and TD and we therefore presented the two parts in the same order. Fatigue may influence the performance data. However, this will affect all participants, both TD and ASD, and we have therefore not adjusted for this in the analyses. Different motivation for the test in the oldest ASD and TD groups could have some influence on the results.

## Conclusion

ASD adolescents between 12 and 16 years showed a tendency to need more time recognizing emotions than TD. In this age group, reaction time and IIV correlated positively with social problems measured by SRS. In adolescents over 16 years, there was no difference between ASD and TD in reaction time, while the IIV correlated negatively with social problems. This resulted in a significant age-dependent interaction between reaction time and reaction time variability and social problems.

The present study suggests a specific cognitive abnormality in ASD that may contribute to the social difficulties and therefore should be investigated in more detail in experimental settings.

## Declarations

### Ethics approval and consent to participate

The study was approved by the Norwegian Regional Committee for Medical and Health Research Ethics South East (2013/1236/REK South-East). Written informed consent was obtained from participants and/or parents when necessary due to age.

## Supporting information

S1 Table Demographics for each diagnostic group. Significance of difference between ASD and TD in right column (*p*-value).
(DOCX)

S2 Table Demographics for each age group. Significance of difference between age groups in right column (*p*-value).
(DOCX)
