# Supplementary material for: The relation between face-emotion recognition and social function in adolescents with autism spectrum disorders: A case control study
Source: PLoS One. 2017 Oct 11;12(10):e0186124. doi: 10.1371/journal.pone.0186124 (PMC5636137; doi:10.1371/journal.pone.0186124)
Supplement: S1 Table — Significance of difference between ASD and TD in right column (p-value). (DOCX) [file pone.0186124.s001.docx]

**S1 Table. Demographics for each diagnostic group. Significance of difference between ASD and TD in right column (*p*-value).**

|  | **ASD** | | **TD** | | | ***p*-value** |
| --- | --- | --- | --- | --- | --- | --- |
|  | n | % | n | | % |  |
|  | 49 | 100 | 49 | | 100 |  |
| **Gender** |  | |  | | |  |
| Male | 36 | 73.5% | 31 | | 63.3% | *p*=0.28 |
| < 16 years | 20 |  | 19 | |  | *p*=0.59 |
| ≥ 16 years | 16 |  | 12 | |  | *p*=0.30 |
| Female | 13 | 26.5% | 18 | | 36.7% |  |
| < 16 years | 6 |  | 8 | |  |  |
| ≥ 16 years | 7 |  | 10 | |  |  |
| **Age – years** | 49 | 100% |  | |  |  |
| Mean (SD); range | 15.6 (±2.4); 11.9-20.9 | | 15.6 (±1.8); 12.3-19.4 | | | *p*=0.95 |
| < 16 years | 26 | 53.1% | 27 | | 55.1% |  |
| Mean (SD); range | 13.7 (±1.3); 11.9-15.7 | | 14.2 (±1.0); 12.3-15.7 | | | *p*=0.09 |
| ≥ 16 years | 23 | 46.9% | 22 | | 44.9% |  |
| Mean (SD); range | 17.8 (±1.3); 16.1-21.0 | | 17.3 (±1.1); 16.1-19.4 | | | *p*=0.12 |
| **SCQ** | 49 | 100% | 47 | 96% | |  |
| Mean (SD); range | 18.7 (±6.7); 5-34 | | 1.9 (±2.3); 0-8 | | | *p*<0.001** |
| < 16 years | 18.3 (±5.9); 6-31 | | 1.5 (±2.2); 0-7 | | | *p*<0.001** |
| ≥ 16 years | 19.1 (±7.6); 5-34 | | 2.5 (±2.4); 0-8 | | | *p*<0.001** |
| **SRS** | 49 | 100% | 48 | 98% | |  |
| Mean (SD); range | 80.1 (±14.4); 47-109 | | 40.6 (±4.2); 34-51 | | | *p*<0.001** |
| < 16 years | 80.1 (±14.6); 54-109 | | 40.0 (±3.3); 35-49 | | | *p*<0.001** |
| ≥ 16 years | 80.2 (±14.4); 47-106 | | 41.3 (±5.1); 34-51 | | | *p*<0.001** |

¹ All but one participant with comorbidity had comorbid AD/HD. These are hence reported twice in the table, both in “More than one comorbidity” and “Comorbid AD/HD”.
