# Supplementary material for: The relation between face-emotion recognition and social function in adolescents with autism spectrum disorders: A case control study
Source: PLoS One. 2017 Oct 11;12(10):e0186124. doi: 10.1371/journal.pone.0186124 (PMC5636137; doi:10.1371/journal.pone.0186124)
Supplement: S2 Table — Significance of difference between age groups in right column (p-value). (DOCX) [file pone.0186124.s002.docx]

**S2 Table. Demographics for each age group. Significance of difference between age groups in right column (*p*-value).**

|  | **< 16 years** | | **≥ 16 years** | | ***p*-value** |
| --- | --- | --- | --- | --- | --- |
|  | n | % | n | % |  |
|  | 53 | 100 | 45 | 100 |  |
| **Gender** |  | |  | |  |
| **Male** | 39 | 58% | 28 | 42% |  |
| ASD | 20 |  | 16 |  | *p*=0.56 |
| TD | 19 |  | 12 |  | *p*=0.23 |
| **Female** | 14 | 45% | 17 | 55% |  |
| ASD | 6 |  | 7 |  |  |
| TD | 8 |  | 10 |  |  |
| **ASD subgroup** |  |  |  |  |  |
| Infantile autism | 5 |  | 8 |  | *p*=0.22 |
| Asperger disorder | 7 |  | 11 |  | *p*=0.13 |
| PDD-NOS | 14 |  | 4 |  | *p*=0.008** |
| **IQ** Mean (SD); range |  | |  | |  |
| Full scale IQ (n=36) | 94.5 (±13.2); 73-127 | | 88.9 (±21.7); 67-133 | | *p*=0.37 |
| Verbal IQ (n=47) | 89.1 (±16.0); 58-117 | | 86.0 (±22.2); 52-130 | | *p*=0.58 |
| Nonverbal IQ (n=48) | 104.7 (±15.1); 73-139 | | 90.4 (±21.1); 58-129 | | *p*=0.009** |
| **Comorbidity** |  | |  |  |  |
| No comorbidity | 15 |  | 16 |  | *p*=0.51 |
| Comorbidity | 11 |  | 7 |  |  |
| More than one comorbidity | 5 |  | 2 |  | *p*=0.34 |
| ADHD/ ADD | 11 |  | 6 |  | *p*=0.23 |
| **SCQ** Mean (SD); range |  | |  | |  |
| ASD | 18.3 (±5.9); 6-31 | | 19.1 (±7.6); 5-34 | | *p*=0.67 |
| TD | 1.5 (±2.2); 0-7 | | 2.5 (±2.4); 0-8 | | *p*=0.16 |
| **SRS** Mean (SD); range |  | |  | |  |
| ASD | 80.1 (±14.6); 54-109 | | 80.2 (±14.4); 47-106 | | *p*=0.99 |
| TD | 40.0 (±3.3); 35-49 | | 41.3 (±5.1); 34-51 | | *p*=0.29 |
